# miR-199a-5p inhibits the expression of ABCB11 in obstructive cholestasis

**DOI:** 10.1016/j.jbc.2021.101400

**Published:** 2021-11-12

**Authors:** Natarajan Balasubramaniyan, Michael W. Devereaux, David J. Orlicky, Ronald J. Sokol, Frederick J. Suchy

**Affiliations:** 1 Department of Pediatrics, Digestive Health Institute, Children’s Hospital Colorado, Aurora, Colorado, USA; 2 Department of Pathology, University of Colorado School of Medicine, Aurora, Colorado, USA

**Keywords:** ATP-dependent efflux transporter, biliary, canalicular, bile acids, cholestasis, microRNA, ABCB11, ATP-binding cassette sub-family B member 11, ABCC2, ATP-binding cassette sub-family C member 2, BSEP, bile salt export pump, FXR, arnesoid X receptor, miR, microRNA, MRP2, multidrug resistance-associated protein 2, Nr1h4, nuclear receptor subfamily 1, group H, member 4, OCA, obeticholic acid, RXR, retinoid X receptor, SMRT, silencing mediator of retinoic acid and thyroid hormone receptor, NCOR1, nuclear receptor corepressor 1

## Abstract

ATP-binding cassette, subfamily B member 11 (ABCB11) is an efflux transporter for bile acids on the liver canalicular membrane. The expression of this transporter is reduced in cholestasis; however, the mechanisms contributing to this reduction are unclear. In this study, we sought to determine whether miR-199a-5p contributes to the depletion of ABCB11/Abcb11 in cholestasis in mice. In a microRNA (miRNA) screen of mouse liver after common bile duct ligation (CBDL), we found that miR-199a-5p was significantly upregulated by approximately fourfold. *In silico* analysis predicted that miR-199a-5p would target the 3′-untranslated region (3′-UTR) of *ABCB11/Abcb11* mRNA. The expression of *ABCB11*-3′-UTR luciferase construct in Huh-7 cells was markedly inhibited by cotransfection of a miRNA-199a-5p mimic, which was reversed by an miRNA-199a-5p mimic inhibitor. We also show treatment of mice after CBDL with the potent nuclear receptor FXR agonist obeticholic acid (OCA) significantly increased *Abcb11* mRNA and protein and decreased miR-199a-5p expression. Computational mapping revealed a well-conserved FXR-binding site (FXRE) in the promoter of the gene encoding miR-199a-5, termed *miR199a-2*. Electromobility shift, chromatin immunoprecipitation, and miR199a-2 promoter-luciferase assays confirmed that this binding site was functional. Finally, CBDL in mice led to depletion of nuclear repressor NcoR1 binding at the *miR199a-2* promoter, which facilitates transcription of *miR199a-2*. In CBDL mice treated with OCA, NcoR1 recruitment to the *miR199a-2* FXRE was maintained at levels found in sham-operated mice. In conclusion, we demonstrate that miR-199a-5p is involved in regulating *ABCB11/Abcb11* expression, is aberrantly upregulated in obstructive cholestasis, and is downregulated by the FXR agonist OCA.

ATP-binding cassette, subfamily B member 11 (ABCB11), also known as the bile salt export pump (BSEP), is an ATP-dependent efflux transporter located exclusively on the liver canalicular membrane (1, 2). The transporter provides the primary motive force for the generation of bile flow and is rate-limiting in the vectorial movement of bile acids from blood to bile (3). A range of congenital and acquired human diseases are associated with malfunction of *ABCB11* (4, 5). The impairmentation of *ABCB11* in humans with cholestasis and various animal models of intrahepatic and obstructive cholestasis lead to retention of bile acids in the hepatocyte and exacerbation of liver injury (6, 7). Depending on the duration, *ABCB11* mRNA and protein levels are depleted in human and experimental cholestasis, but the mechanisms underlying these changes have not been completely characterized (5, 8).

The nuclear receptor FXR plays a central role in the regulation of bile acid synthesis and transport in the liver and intestine; and several FXR agonists are being used or under study for the treatment of cholestatic disorders (9, 10). The expression of bile salt export pump (BSEP) depends on transactivation by FXR in health and disease (11). Common bile duct ligation (CBDL) in FXR knockout mice leads to undetectable hepatic BSEP expression and much more severe hepatic injury compared with wild-type mice (12). FXR agonists provide hepatoprotection in rodent models of intra- and extrahepatic cholestasis (13). The signaling pathways through which FXR acts to improve the cholestatic phenotype have not been completely defined.

There are complex regulatory mechanisms that influence expression and function of ABCB11 and other transporters in health and disease (14, 15). Normal cellular processes are subject in part to regulation by miRNAs, and alterations in miRNA expression or function can contribute to the pathogenesis of disease in animal models and humans (15, 16, 17). MicroRNAs (miRNA) are a family of small noncoding RNA molecules (containing ∼22 nucleotides) that act in RNA silencing and posttranscriptional regulation of gene expression by base pairing to sequence motifs in the 3′ UTR of target mRNAs (18). The human genome encodes approximately 2600 mature miRNAs (miRbase v.22), and more than 200,000 transcripts, including isoforms with slight variation, are annotated in GENECODE (v.29) (19, 20). An increasing number of target sites for miRNAs are being validated within 3′UTRs of human mRNAs using reporter gene assays and demonstration of microRNA-mRNA interaction *via* techniques such as cross-linking immunoprecipitation (CLIP) (19, 20).

In a previous miRNA screen of mouse liver after biliary obstruction, we found that multiple miRNAs were significantly upregulated or downregulated (21). In the current study we confirmed that miR-199a-5p was significantly upregulated in this model, and bioinformatic analysis predicted that miR-199a-5p would target the 3′ UTR of *ABCB11*. *In vitro* and *in vivo* studies confirmed that this miRNA targeted the 3′ UTR of *ABCB11* RNA. Moreover, the expression of miR-199a-5p was suppressed by treatment of mice after CBDL with the potent FXR agonist obeticholic acid (OCA) (6α−ethyl-chenodeoxycholic acid). The goal of this study was to determine the functional importance of miR199a-5p in regulating ABCB11 and how FXR agonism alters miR-199a-5p expression in a liver cell line and cholestatic mice.

## Results

### The effect of common bile duct ligation on expression of ABCB11/Abcb11 and miR-199a-5p

**Figure 1:**
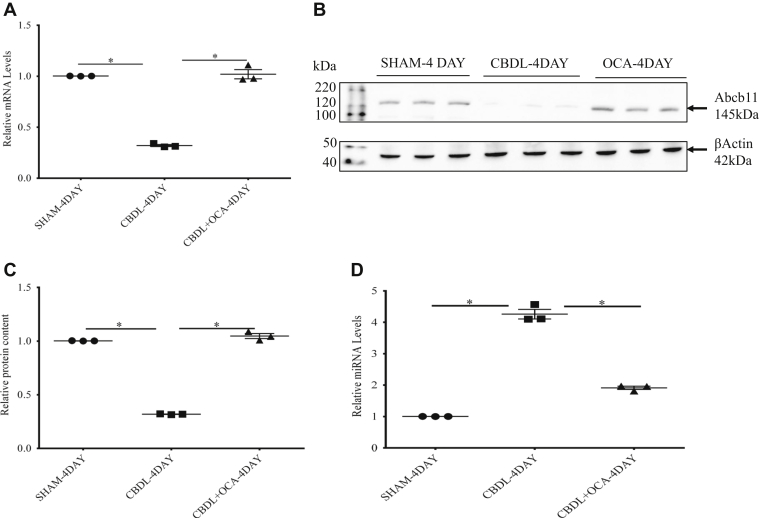
**The effect of CBDL on expression of *Abcb11* mRNA and miR-199a-5p in mice with and without treatment with obeticholic acid (OCA).***A*, four days of CBDL led to significant depletion of *Abcb11* mRNA and (*B* and *C*) Abcb11 protein levels on Western blot and quantification analysis, respectively in CBDL *versus* sham-operated mice. *D*, upregulation miR-199a-5p in CBDL *versus* sham-operated mice by RT-PCR analysis. *A*–*D*, after 4 days of CBDL in mice treatment with obeticholic acid preserved *Abcb11* mRNA and protein and suppressed miR-199a-5a expression at levels found in sham operated. Experiments were repeated three times. Statistical analysis was performed using one-way analysis of variance and Tukey’s correction for multiple comparisons. ∗ *p* ≤ 0.05.

As has been reported, *Abcb11* mRNA and protein were significantly depleted after 4 days of common bile duct obstruction (CBDL) in mice, which is confirmed in Figure 1, *A*–*C* (1). We have previously used a Mouse Liver miFinder miScript miRNA PCR Array from Qiagen to study the expression of miRNAs abundantly expressed or best characterized in liver tissue after biliary obstruction (21). Among numerous miRNAs that were significantly up- or downregulated in the mouse after complete CBDL, miR-199a-5p was upregulated approximately fourfold compared with shame-operated mice. This result was confirmed by RT-PCR analysis (Fig. 1*D*) in which a fourfold increase in miR-199a-5p was also demonstrated. The changes in *ABCB11* and miR-199a-5p expression persisted at the same levels in mice studied at 7 and 14 days after CBDL (Fig. S1).

After 4 days of CBDL in mice treatment with OCA preserved *Abcb11* mRNA and protein levels and suppressed miR-199a-5a expression at levels found in sham operated mice (Fig. 1, *A*, *B*, and *D*).

In mice treated with OCA, there was also significant attenuation of liver injury as reflected in lower serum aminotransferases, total bilirubin and decreases in areas of hepatocyte necrosis compared with untreated mice (Fig. S2 and Table S1).

**Figure 2:**
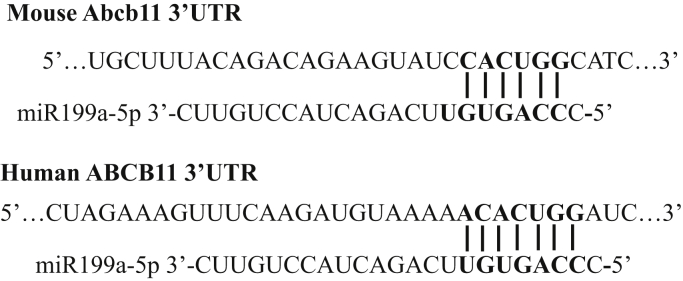
**Predicted binding sites for miR 199a-5p in 3′ untranslated regions of the mouse (*Abcb11*) and human (*ABCB11*) mRNAs.***In silico* analysis using miRANDA, DIANA-microT-CDS, and miRBase microRNA target prediction algorithms all identified a potential miR-199a-5p binding site in the 3′UTR of *Abcb11* and *ABCB11* mRNAs. The alignments of miR-199a-5p with mouse *Abcb11* and human *ABCB11* 3′ UTRs are shown above.

*In silico* analysis using miRANDA, DIANA-microT-CDS, and miRBase microRNA target prediction algorithms all identified a potential miR-199a-5p binding site in the 3′UTR of *Abcb11* and *ABCB11* mRNAs. The alignments of miR-199a-5p with mouse *Abcb11* and human *ABCB11* 3′ UTRs are shown in (Fig. 2).

### Expression of ABCB11 and miR-199a-5p infants with biliary atresia

**Figure 3:**
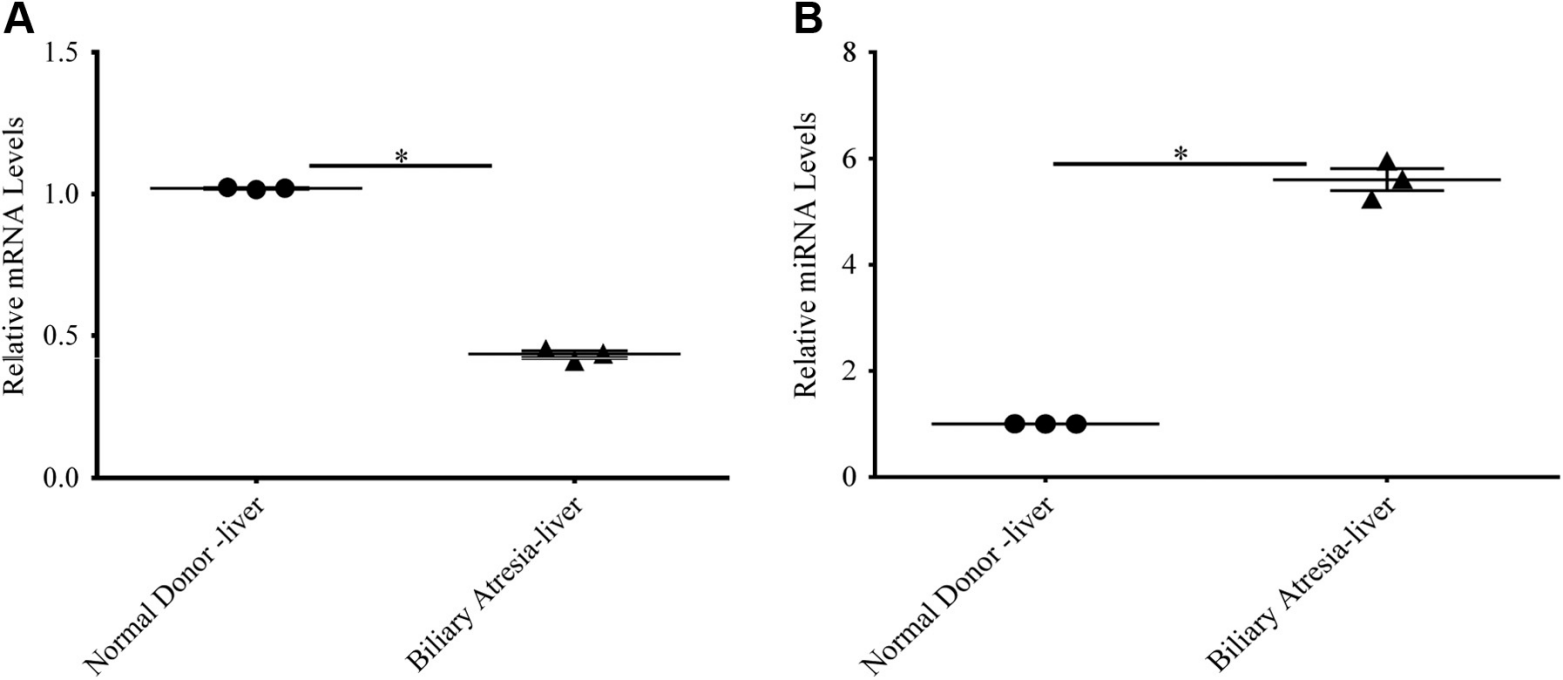
**The expression of ABCB11 and miR-199a-5p in infants with biliary atresia.***A*, *ABCB11* mRNA was significantly depleted and (*B*) miR-199a-5p was elevated in livers of infants with biliary atresia by RT-PCR at the time of Kasai portoenterostomy compared with normal livers (mean of three livers in each group). A two-tailed paired Student *t* test was used in comparing two groups. ∗ *p* ≤ 0.05.

*ABCB11* mRNA was also significantly depleted and miR-199a-5p was elevated over fourfold in livers of infants with biliary atresia at the time of Kasai portoenterostomy compared with normal livers (Fig. 3, *A* and *B*). The downregulation of *ABCB11* early in the course of biliary atresia agrees with a previous study (5).

### A functional miR-199a-5p responsive element is present in the 3′UTR of ABCB11 mRNA

**Figure 4:**
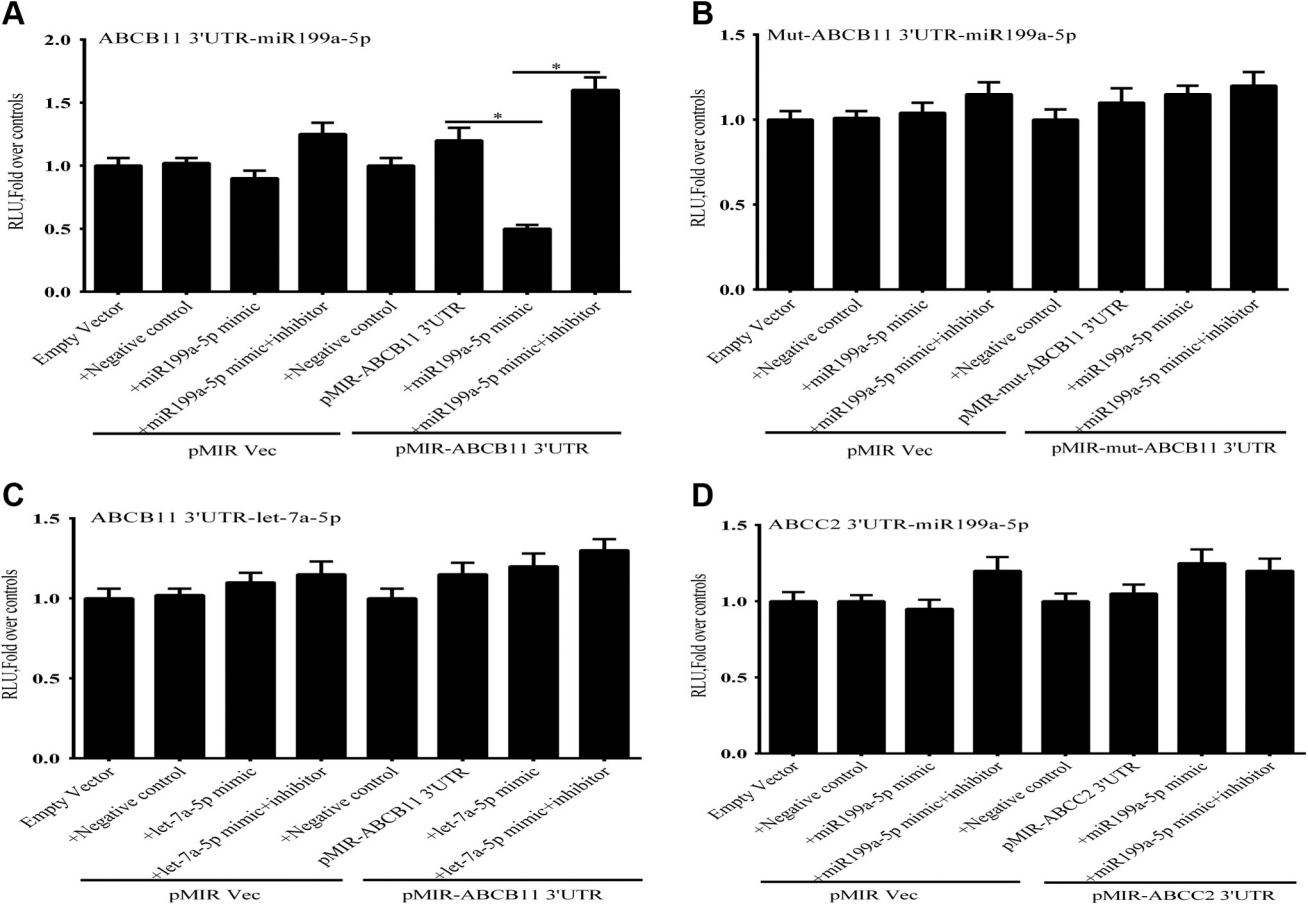
**miR-199a-5p regulates the expression of *ABCB11* by targeting the 3′-UTR of its mRNA.** Huh7 cells were transfected with an *ABCB11*-3′ UTR-luciferase construct in the presence of a miR-199a-5p mimic with and without a mimic inhibitor. Luciferase activity was measured 48 h after transfection. *A*, expression of a miR-199a-5p mimic significantly repressed luciferase expression. The activity of the mimic was completely reversed by a mimic inhibitor. *B*, site-directed mutagenesis of the miR-199a-5p binding site in the *ABCB11*-3′ UTR completely abrogated the inhibitory effect of the miR-199a-5p mimic on luciferase expression. *C*, a miR-let7a-5p mimic had no effect on expression of an *ABCB11*-3 UTR-luciferase construct expressed in Huh7 cells. *D*, a miR199-5p mimic did not suppress expression of an *ABCC2*-3′UTR -luciferase construct expressed in Huh-7 cells. Statistical analysis was performed using one-way analysis of variance and Tukey’s correction for multiple comparisons. Each panel represents the mean ± SEM of three separate experiments. ∗ *p* ≤ 0.05.

The 3′ untranslated region (UTR) of *ABCB11* mRNA was cloned immediately downstream of a *Renilla* luciferase reporter in a pRL-CMV plasmid. Cotransfection of a miR-199a-5p mimic and the *ABCB11* 3′ UTR-luciferase construct into Huh-7 cells led to a marked inhibition of luciferase activity by ∼60 to 70% compared with controls (Fig. 4*A*), which was reversed by a miR-199a-5p mimic inhibitor. Site-directed mutagenesis of the miR-199a-5p binding site in the *ABCB11-3′* UTR blocked the inhibitory effect of the miR-199a-5p mimic on luciferase expression (Fig. 4*B*). We have previously shown that microRNA Let7a-5p was also upregulated approximately fourfold after CBDL and targets the 3′-UTR of *ABCC2 (MRP2)* (21), but overexpression of a mimic for this miRNA had no effect on expression of the *ABCB11* -3′ UTR-luciferase construct, which lacks a binding site for this miRNA (Fig. 4*C*). Moreover, an miR199-5p mimic did not suppress expression of an *ABCC2*-3′UTR-luciferase construct in Huh-7 cells (Fig. 4*D*).

### Expression of a 199a-5p mimic in Huh-7 cells leads to depletion of *ABCB11* mRNA and protein.

**Figure 5:**
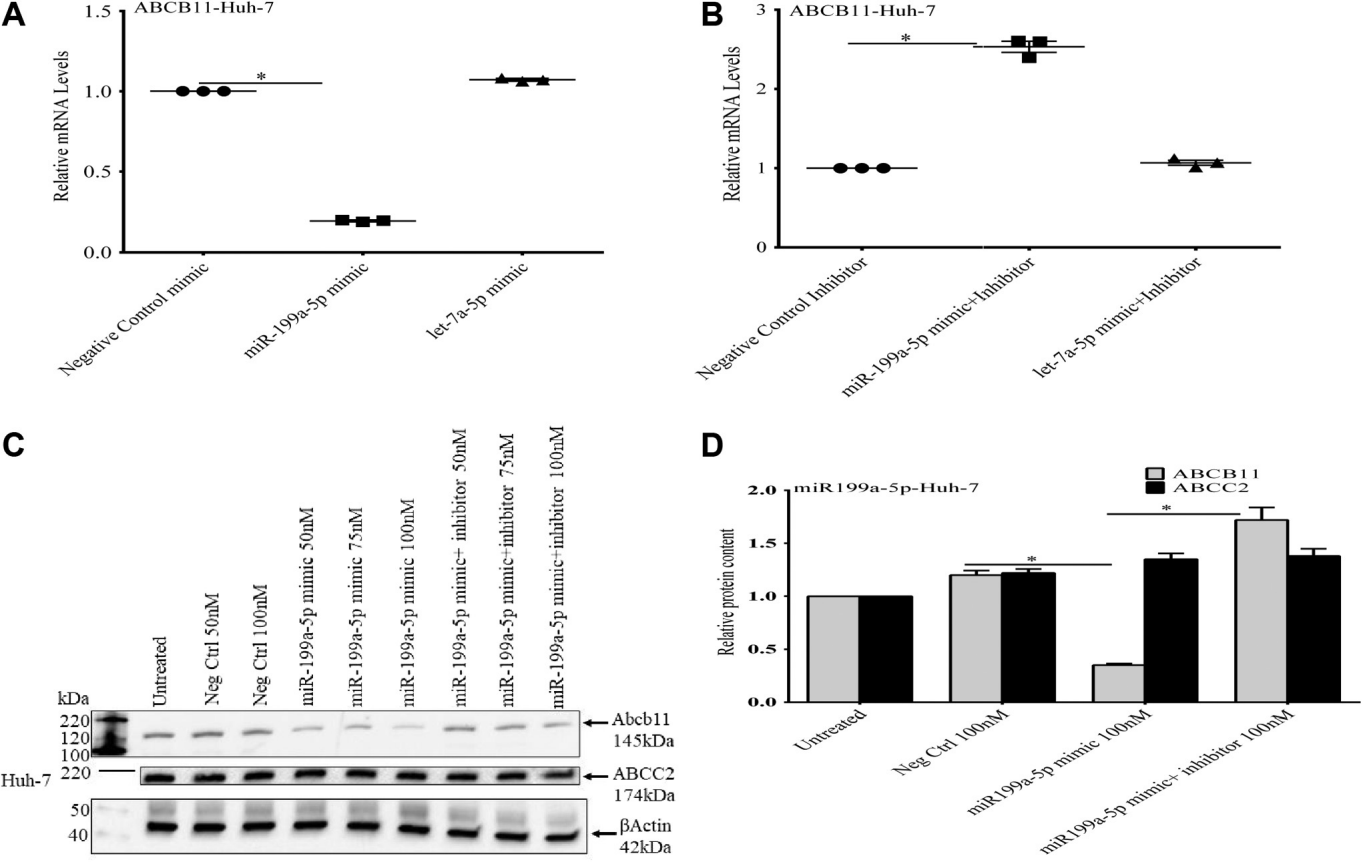
**The effect of an miR-199a-5p mimic on endogenous *ABCB11* mRNA and protein levels in Huh-7 cells.***A*, transfection of an miR-199a-5p mimic but not a let7a-5p mimic into Huh-7 cells led to a significant decrease in *ABCB11* mRNA levels. *B*, the effect of a miR-199a-5p mimic was blocked by expression of a miR-199a-5p mimic inhibitor. *C*, the miR-199a-5p mimic also suppressed endogenous ABCB11 protein levels. *D*, the miR-199a-5p mimic had no effect on ABCC2 protein levels in Huh-7 cells. Statistical analysis was performed using one-way analysis of variance and Tukey’s correction for multiple comparisons. Each panel represents the mean ± SEM of three separate experiments. ∗ *p* ≤ 0.05.

To investigate the effects of miR-199a-5p on endogenous ABCB11 expression, Huh-7 cells were transfected with a miR-199a-5p mimic. Overexpression of a miR-199a-5p mimic but not a miR-let7a-5p mimic led to a significant decrease in *ABCB11* mRNA levels (Fig. 5*A*). Overexpression of an miR-199a-5p mimic inhibitor blocked the inhibitory effect of the miR-199a-5p mimic and led to an increase in endogenous *ABCB11* mRNA levels (Fig. 5*B*). In contrast, expression of a miR-let7a-5p mimic had no effect on *ABCB11* RNA levels and an miR-199a-5p mimic had no effect on levels of *ABCC2 m*RNA levels (not shown). Expression of an miR-199a-5p mimic also markedly suppressed ABCB11 protein levels in Huh-7 cells but had no effect on the amounts of ABCC2 protein levels (Fig. 5*C*). This effect was blocked by cotransfection of a miR-199a-5p mimic inhibitor (Fig. 5, *C* and *D*).

### The effect of FXR on miR199a-5p and Abcb11 expression *in vitro* and *in vivo*

**Figure 6:**
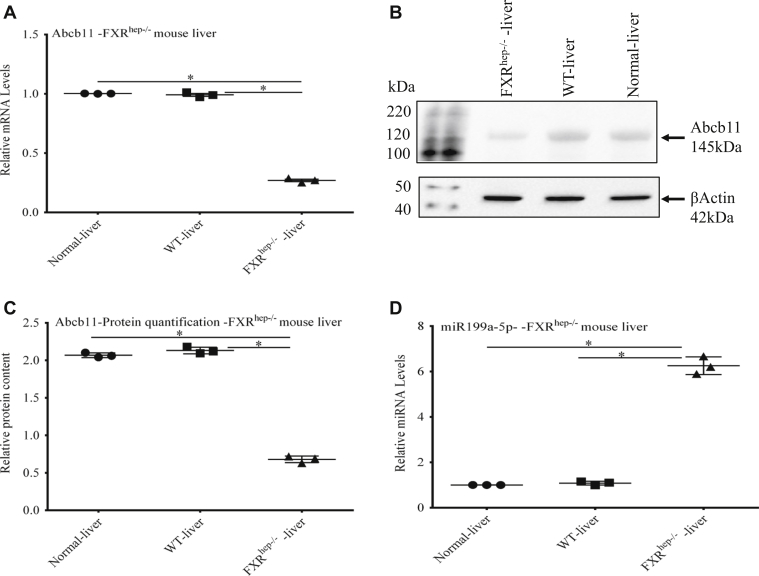
**Expression of *ABCB11* and miR 199a-5p in FXR hepatocyte-specific knockout mice.***A*, *Abcb11* mRNA and (*B* and *C*) protein levels were markedly depleted in livers from FXR hepatocyte-specific knockout mice (FXR^hep−/−^) compared with normal mouse livers. *D*, there was increased expression of miR199a-5p in the FXR^hep−/−^ mice. Statistical analysis was performed using one-way analysis of variance and Tukey’s correction for multiple comparisons. Each panel represents the mean ± SEM of three separate experiments. ∗ *p* ≤ 0.05.

Analysis of liver tissue from FXR hepatocyte-specific knockout mice (FXR^hep−/−^) provided additional evidence about the role of FXR in regulating Abcb11 and miR199a-5p expression. As expected, (Fig. 6) *Abcb11* mRNA and Abcb11 protein levels were depleted by ∼75% and miR199a-5p expression was increased approximately sixfold in these mice.

**Figure 7:**
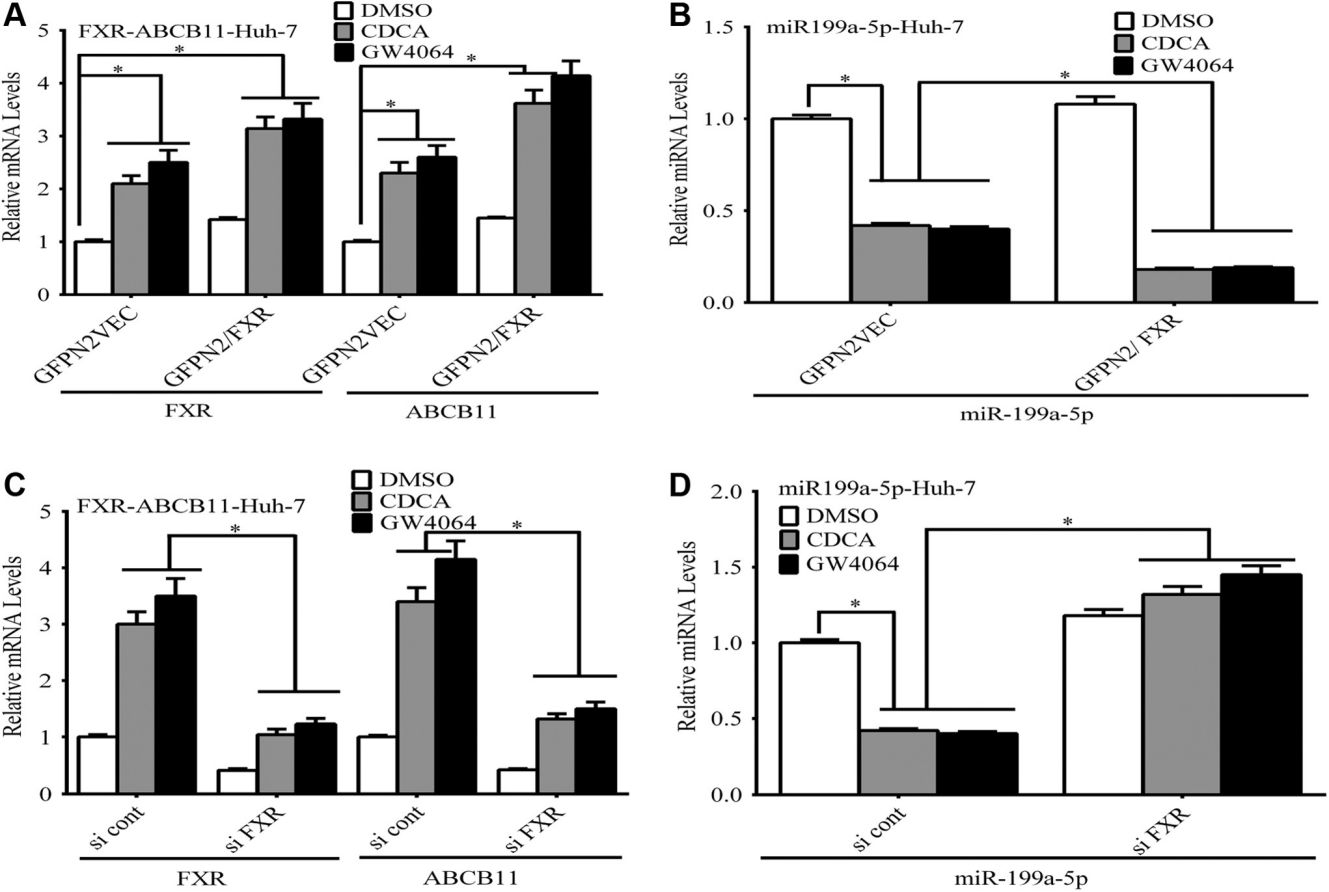
**The effect of FXR agonists on expression of *ABCB11* and miR-199a-5p in Huh-7 cells.***A*, overexpression of FXR in Huh-7 cells in the presence of the FXR agonist GW4064 or chenodeoxycholic acid led to marked induction of *ABCB11* mRNA and (*B*) suppression of *miR199a-5p* mRNA by RT-PCR analysis. *C*, siRNA knockdown of FXR expression in the presence of the FXR agonist GW4064 or chenodeoxycholic acid suppressed *ABCB11* mRNA and (*D*) increased *miR-199a-5p* mRNA levels in these cells. Statistical analysis was performed using one-way analysis of variance and Tukey’s correction for multiple comparisons. Each panel represents the mean ± SEM of three separate experiments. ∗ *p* ≤ 0.05.

Studies were then done *in vitro* to define the effects FXR agonists on expression of ABCB11/Abcb11 shown in Figure 1. First, overexpression of FXR in Huh-7 cells in the presence of the FXR agonists GW4064 or chenodeoxycholic acid led to marked induction of *ABCB11* mRNA and suppression of *miR199a-5p* mRNA (Fig. 7, *A* and *B*). Conversely, siRNA knockdown of FXR expression in the presence of the GW4064 or chenodeoxycholic acid suppressed *ABCB11* mRNA and increased *miR-199a-5p* mRNA levels in these cells (Fig. 7, *C* and *D*).

### An FXR-binding site (FXRE) is present in the promoter of the gene encoding miR-199a-5

**Figure 8:**
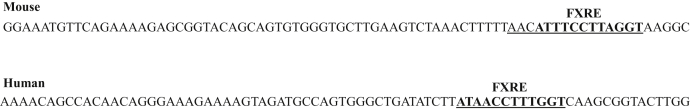
**A FXR-binding site (FXRE) in the mouse and human *miR199a-2* promoters.** A potential FXR-binding site (FXRE) was identified in the promoter of the gene encoding miR-199a-5, termed *miR199*a*-2*, in the human and mouse using the bioinformatic tools.

To further define the mechanisms underlying *in vitro* and *in vivo* effects of FXR agonists on miR-199a-5p expression, a bioinformatic analysis was done that revealed a well-conserved FXR-binding site (FXRE) in the promoter of the gene encoding miR-199a-5p, termed *miR199a-2*. These binding sites were identified ∼ -1150 and -800 base pairs from the transcription start sites in the human and mouse genes, respectively. The sequence for this site in both the mouse and human genes is shown in Figure 8.

Two methods were used that demonstrated specific FXR binding to FXR-binding site (FXRE) in the promoter of *miR199a-2*.

**Figure 9:**
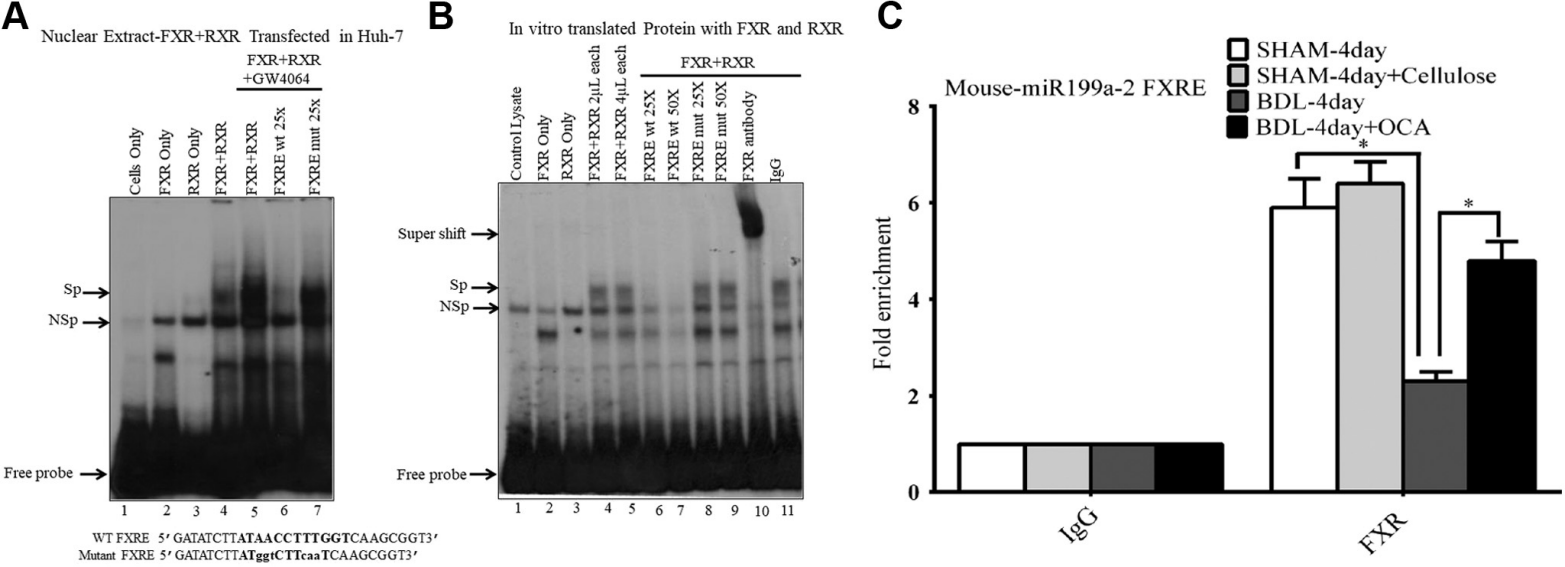
**Analysis of the putative FXRE in the promoter of *miR199a-2*, the gene encoding miR-199a-5p.** EMSAs were carried out as described under “Experimental procedures” using a ^32^P-labeled human *miR199a-2* promoter FXRE. First Huh-7 cells were transfected with an expression plasmid for human FXR and RXRα. *A*, enhanced specific binding of FXR/RXR to the FXRE in the *miR199a-2* promoter occurred in the presence of the FXR agonist GW4064 (lane 5). FXRE binding was specifically competed off by the addition of a 25-fold molar excess of *miR199a-2* promoter FXRE wild-type oligo (lane 6), but not by an FXRE mutant oligonucleotide (lane 7), Sp, specific DNA–protein complex; NSp, nonspecific complex. DNA sequence of the wild-type (WT) and mutant FXRE shown with the mutated residues indicated in *lowercase* type. *B*, *in vitro* translated FXR and RXRα proteins were used in EMSA with *miR199A-2* FXRE as a probe as described under “Experimental procedures.” Unprogrammed lysate (lane 1) and lysates programmed with expression plasmid FXR (lane 2), RXRα (lane 3), or FXR/RXRα (lanes 4–5) translated protein were incubated with *miR199A-2* FXRE probe. Competition analysis was performed with unlabeled 25 and 50-fold molar excess of wild type (lane 6–7) or mutant FXRE (lane 8–9). A super-shifted band was demonstrated with the addition of an FXR antibody (lane 10). Lane 11 shows incubation with IgG control. Sp, specific complex; NSp, nonspecific complex. *C*, *in vivo* Chromatin immunoprecipitation (ChIP) experiments were done on liver homogenates from mice after CBDL, CBDL+obeticholic acid treatment mice, SHAM-operated mice, and SHAM-operated mice +Cellulose. Specific binding of FXR to the promoter of mouse *miR199a-2* was demonstrated by quantitative PCR data with specific primers. Depletion of FXR is shown at *miR199A-2* FXRE after CBDL compared with sham-operated mice. Treatment of CBDL mice with obeticholic acid increased recruitment of FXR to the *miR199A-2* FXRE by over twofold. Data presented as fold change over IgG control. ∗, *p* < 0.05 *versus* all other treatments using one-way ANOVA and Tukey's correction.

First, an electromobility shift assay (EMSA) was done using nuclear extracts prepared from Huh-7 cells transfected with FXR and RXRα (Fig. 9*A*). The EMSA demonstrated the specific interaction between the FXR/RXRα and a ^32^P-labeled *miR199a-2* promoter FXRE probe (Fig. 9*A*). The addition of nuclear extract from FXR/RXR-transfected Huh-7 cells led to the appearance of a shifted DNA-protein complex (lane 4), but a shifted band was not observed in lanes with extracts from cells without transfections (lane 1) or from cells transfected with FXR (lane 2) or RXR (lane 3) alone. Enhanced specific binding of FXR/RXR to the FXRE in the *miR199a-2* promoter occurred in the presence of the FXR agonist GW4064 (lane 5). FXRE binding was specifically competed off by the addition of a 25-fold molar excess of *miR-199a-2* promoter FXRE wild-type oligo (lane 6), but not by an FXRE mutant oligonucleotide (lane 7), demonstrating the specificity of the complex.

Next, to investigate whether the formation of the DNA protein complex requires the presence of the FXR/RXRα heterodimer, EMSA was repeated using *in vitro* translated FXR and RXRα proteins as shown in Figure 9*B*. Neither unprogrammed lysate nor expression of FXR or RXRα alone (Figure lanes 1–3, respectively) bound to *miR-199a-2* FXRE probe. However, when both FXR and RXRα were incubated together, a complex was formed, indicating that FXR/RXR heterodimer is bound (Fig. 9*B* lanes 4 and 5, Sp arrow) by the *miR199a-2* promoter IR-1 element. The binding was specific, as demonstrated by competition with an excess (25 and 50-fold) of FXRE oligonucleotide (lanes 6, 7) but not a mutated oligonucleotide (lanes 8, 9). A super-shifted band was demonstrated with the addition of an FXR antibody (lane 10).

Chromatin immunoprecipitation (ChIP) analysis was next used to explore interactions between mouse and FXR to the *miR199a-2* promoter FXRE within the natural chromatin context of the cell. *In vivo* ChIP analysis of the *miR199a-*2 promoter was done using mouse liver after 4 days of CBDL, after sham surgery (Fig. 9*C*) and after treatment with OCA. There was marked depletion of FXR binding at *miR199a-2* FXRE after CBDL compared with sham-operated mice, a change that would enhance the inhibitory effects of this microRNA on ABCB11 expression. Treatment of CBDL mice with OCA increased recruitment of FXR to the *miR199a-2* FXRE by over twofold.

**Figure 10:**
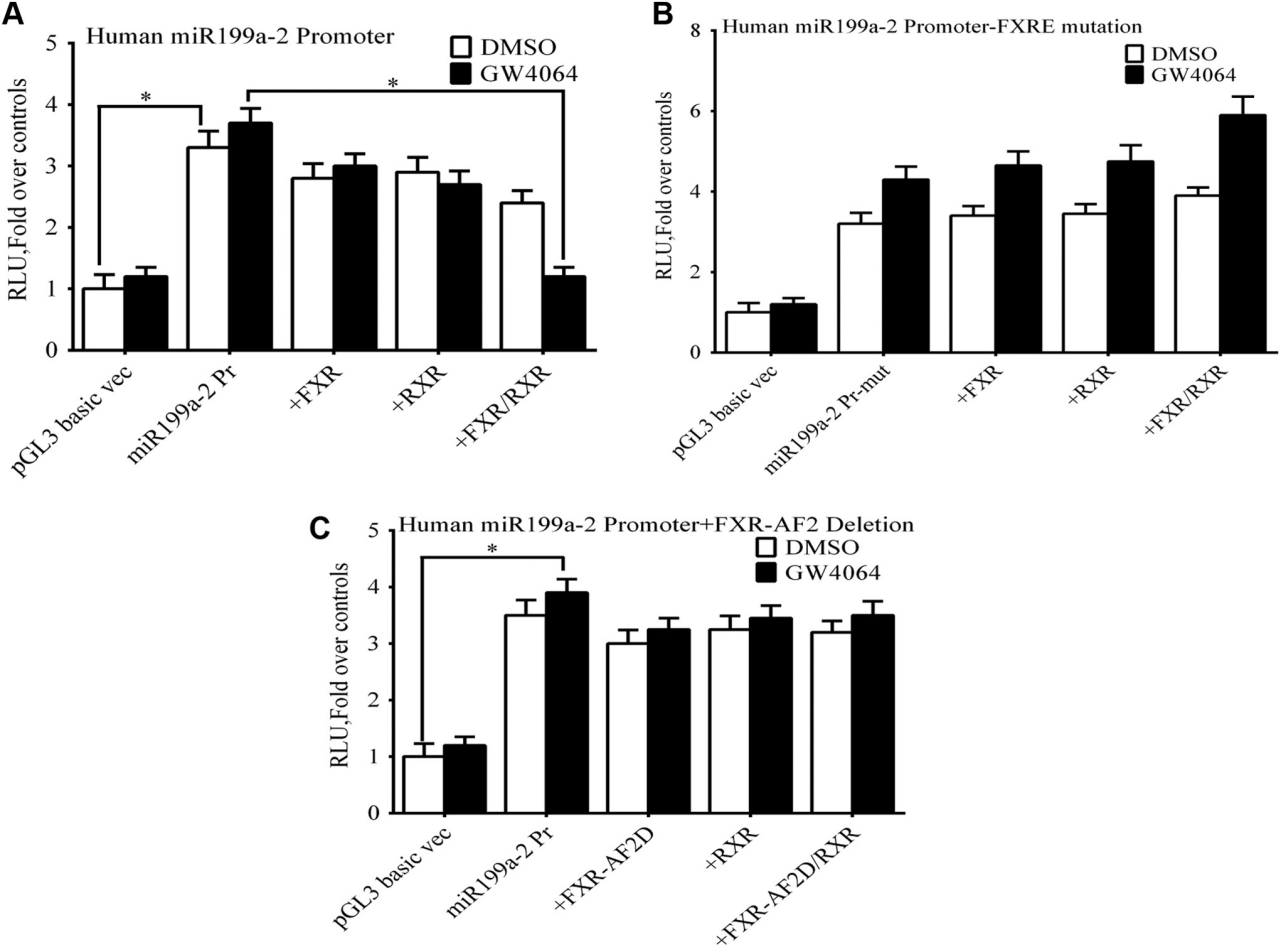
**The effect of FXR/RXR expression on the activity of the *miR199a-2* promoter.** Huh-7 cells transfected with indicated plasmids were treated with the FXR ligand GW 4064 24 h after transfection, and reporter assays were performed. Luciferase activity was assayed in the presence and absence of ligands and normalized to β-galactosidase luciferase. *A*, FXR/RXR expression significantly reduced *miR199a-2* reporter gene activity in the presence of GW4064. *B*, mutation of the FXRE in the *miR199a-2* promoter blocked the inhibitory effects of FXR/RXR on expression m*iR199a-2* reporter gene activity in the presence of GW4064 (*C*) A AF-2 deletion mutant of FXRh also failed to transactivate *miR199a-2* Luc promoter. Data presented as fold change over *miR199a-2* promoter only. ∗, *p* < 0.05 *versus* all other treatments using one-way ANOVA and Tukey's correction. The mean and mean ± SE are shown (n = 3).

Next, the *miR199a-2* promoter was cloned immediately downstream of a luciferase reporter and expressed in Huh-7 cells. Transfection of FXR/RXR in these cells markedly inhibited luciferase activity when incubated with the FXR agonist GW4064 compared with addition of a DMSO control or when FXR or RXR was expressed alone (Fig. 10*A*). Mutation of the FXRE in the *miR199a-2* promoter blocked the inhibitory effects of FXR/RXR expression in the presence of GW4064 (Fig. 10*B*). Deletion of the AF2 (ligand-binding domain) domain of FXR (Fig. 10*C*) completely abrogated the inhibitory effects of the FXR agonist GW4064 on *miR199a-2* promoter. These studies indicate that OCA improves expression of ABCB11 by a novel mechanism in which FXR agonism blocks the inhibitory effects of miR199a-5p.

**Figure 11:**
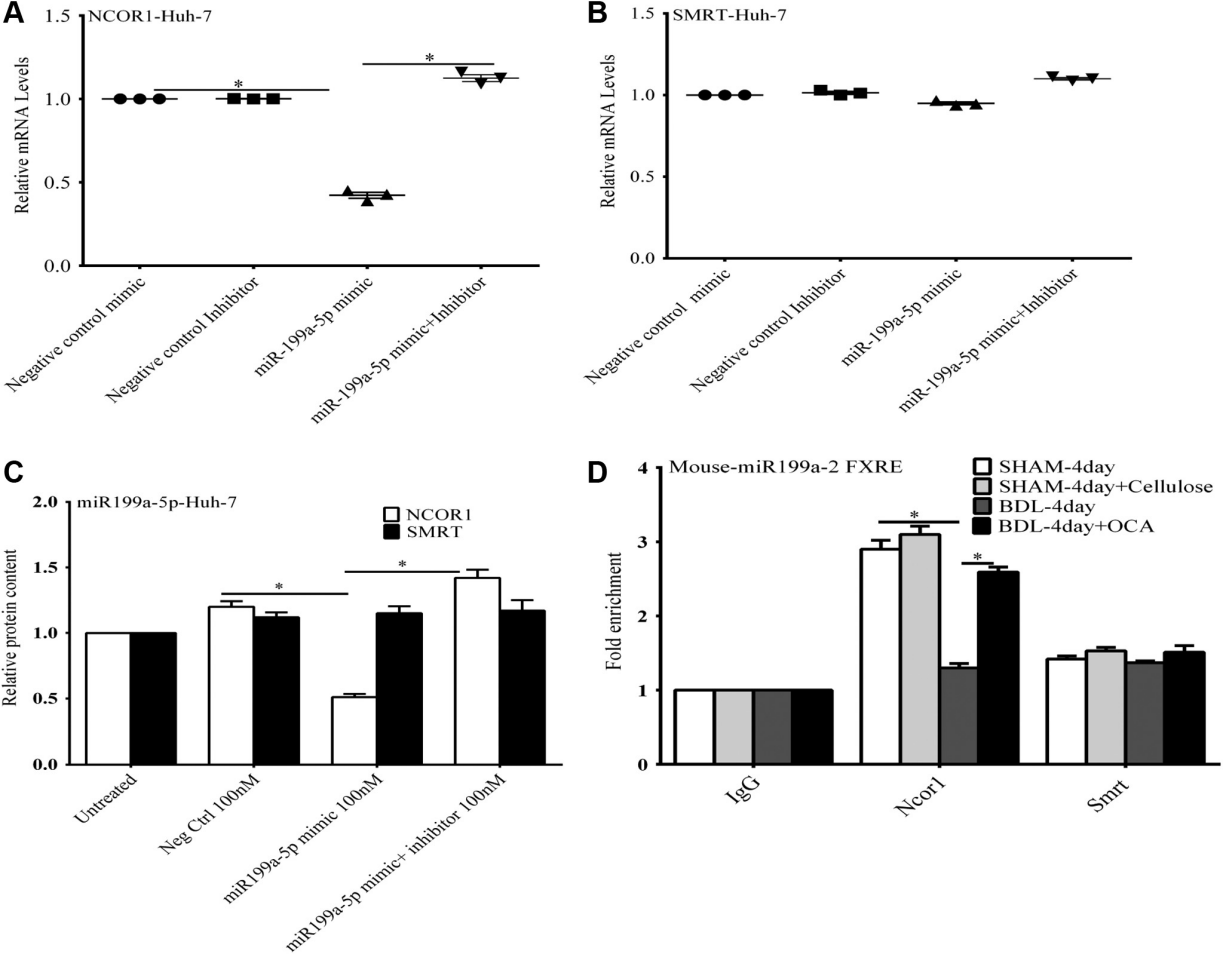
**The effect of nuclear receptor corepressors on expression of miR-199a-5p.***A* and *C*, transfection of a miR-199a-5p mimic into Huh-7 cells led to a significant decrease *in NCoR1* mRNA and protein levels, which could be blocked by expression of an miR-199a-5p mimic inhibitor. *B* and *C*, the expression of the miR-199a-5p mimic had no effect of *SMRT* mRNA and protein levels. *D*, *in vivo* ChIP analysis of the *miR199a-2* promoter was done using mouse liver after common bile duct ligation (CBDL), after sham surgery, and after treatment with obeticholic acid. There was marked depletion of NcoR1 binding at *miR199a-*2 FXRE after 4 days of CBDL compared with sham-operated mice as determined by quantitative RT-PCR. In mice treated with OCA NcoR1 recruitment to the FXRE was maintained at levels found in sham-operated mice. There was no change in recruitment of a related corepressor, SMRT, under any of the experimental conditions. ∗, *p* < 0.05 *versus* all other treatments using one-way ANOVA and Tukey's correction. Each panel represents the mean ± SEM of three separate experiments.

Nuclear receptors such as FXR repress or stimulate transcription by recruiting corepressor or coactivator proteins to regulated promoters. NCoR1 (nuclear receptor corepressor) and SMRT (silencing mediator of retinoic acid and thyroid hormone receptor) are among the best-characterized corepressors that have been shown to bind to a wide range of unliganded nuclear receptors (22). NCoR1 and SMRT serve as scaffolding proteins facilitating the formation of large transcriptional corepressor complexes that include histone deacetylases. Expression of an miR-199a-5p mimic (Fig. 11, *A*–*C*) in Huh-7 cells suppressed NCoR1 mRNA and protein levels, but had no effect on SMRT expression.

*In vivo* ChIP analysis of the *miR199a-2* promoter was done using mouse liver after 4 days of CBDL, after sham surgery and after treatment with OCA (Fig. 11*D*). There was marked depletion of NcoR1 binding at *miR199a-2* FXRE after 4 days of CBDL compared with sham-operated mice as determined by quantitative RT-PCR, a change that would facilitate of transcription of *miR199a-2*. In mice treated with OCA NcoR1 recruitment to the FXRE was maintained at levels found in sham-operated mice. There was no change in recruitment of a related corepressor, SMRT, under any of the experimental conditions, confirming the specificity of NcoR1 effects in this model.

## Discussion

The bile salt export pump (BSEP/ABCB11) belongs to the ATP-Binding Cassette (ABC) protein family and is a critical determinant of the enterohepatic circulation of the bile salts (7). We have previously shown that expression of *ABCB11* is regulated by the farnesoid X receptor (FXR), a bile acid sensor, which forms a heterodimer with RXRα upon activation, and is bound to an FXR response element in the promoter of *ABCB11* (11). We and others have also found and shown that a number of other nuclear receptor coactivating proteins contribute FXR-mediated transcriptional activation including the Set7/9 methyltransferase (23), components of the activating signal cointegrator-2-containing complex (ASCOM) (24), the coactivator-associated arginine methyltransferase 1 (CARM1) (25), and the steroid receptor coactivator 1 (SRC2) (26). The coactivators attracted to promoters of FXR target genes such as *ABCB11* and the epigenetic modifications that occur after ligand binding to FXR have not been completely defined, and it is unknown whether and to what extent these processes are disrupted during cholestasis and contribute to the disease process. miRNAs provide another level of epigenetic regulation that could act synergistically with these mediators in health and disease. Efflux pumps of the ABC (ATP-binding cassette) transporter family are also subject to miRNA-mediated gene regulation (27). In a previous study we found that miR-33 targeted the 3′-UTR of *ABCB11/Abcb11* and several other canalicular membrane transporters and was upregulated in mice treated with simvastatin and/or a lithogenic diet (28). In our screen of miRNAs after CBDL miR-33 expression was not altered (21), indicating that different miRNAs may be induced or depleted depending on the pathophysiological context.

This study demonstrates that miR199a-5p regulates the expression of *Abcb11* in normal and cholestatic mouse liver. miR-199a-5p was found to be upregulated over fourfold after common duct ligation. Multiple in *silico* methods predicted that miR199a-5p would target the 3′UTR of *ABCB11/Abcb11*. *In vitro* experiments then demonstrated that expression of an miR199a-5p mimic depressed Abcb11 mRNA and protein levels as well the activity of a *ABCB11*-3′ UTR-luciferase construct in Huh-7 cells. Transfection of a mimic for another microRNA, miR-let7a-5p, also upregulated in obstructive cholestasis, had no effect on ABCB11 expression.

The expression of *ABCB11/Abcb11* is variably altered in various forms of experimental and human cholestasis and is dependent on etiology and duration (2, 29). Expression of Abcb11 was initially decreased in several rodent models after endotoxin or ethinylestradiol exposure and after CBDL, but recovery may occur with prolonged cholestasis (8, 29, 30). Similarly, as confirmed in this study, early in the course of biliary atresia in infants, most canalicular transporters are downregulated, including bile salt export pump (BSEP, ABCB11), multidrug-resistant protein 3 (MDR3, ABCB4), and the multidrug-resistant associated protein 2 (MRP2, ABCC2) (5). Partial recover occurs with prolonged cholestasis. In contrast, in adult patients with primary sclerosing cholangitis, *ABCB11* was significantly overexpressed compared with other cholestatic and noncholestatic liver diseases, but these were patients with well-established liver disease (31). The decreased expression of *ABCB11* in complete biliary obstruction could be beneficial, as continued transport of bile acids further may damage hepatocytes in an obstructed biliary tree. In cholestatic conditions, *ABCC4* (MRP4) localized to the basolateral membrane is upregulated and may become a key compensatory pathway for efflux of bile acids from hepatocytes into blood (29, 32).

MicroRNAs target specific mRNAs for degradation or translational repression, thereby influencing cell cycle progression, proliferation, differentiation, apoptosis, organogenesis, and maintenance of organ function. The mRNA and miRNA regulatory network is complicated in that each miRNA can potentially target many mRNAs, and each mRNA can potentially have a large number of miRNA binding sites in its 3′-UTR (18). How multiple miRNAs targeting the same mRNA are utilized is not well understood, but may depend on the cellular context including disease states. Dysregulation of miRNAs may disrupt gene regulatory networks and contribute to the pathogenesis of disease (15, 16).

After a candidate miRNA for a disease process has been suggested based on miRNA arrays and bioinformatic analysis, the challenge remains to identify and validate the physiologic target mRNA(s) (20). *In vitro* miRNA overexpression or antagonism along with functional and cellular mRNA analysis is essential in identifying miRNA-targeted genes. As was done in this study, correlation of an miRNA level with expression in animal models and in human disease including a response to a therapeutic intervention provides another level of validation. However, in contrast to the dramatic changes observed with *in vitro* overexpression of miR-199a-5p, this miRNA is likely to have a more modest phenotypic effect *in vivo*, acting in concert with other transcriptional regulators including other miRNAs.

miR-199a-5p is one of the most abundantly expressed microRNAs in the liver (33, 34). Many miRNAs that are identified as potentially important in disease states may not be contained in the miRNA databases and require extensive validation as was done in the current study (35). The MicroRNA Target Prediction Database (http://mirdb.org) has identified 562 predicted targets for miR-199a-5p, but did not include *ABCB11/Abcb11*. As part of a previous survey of microRNAs during obstructive cholestasis, miR-199a-5p has been shown to be upregulated in the liver of bile-duct-ligated mice, but the functional role in this model was not evaluated (36). miR-199a-5p is also elevated following bile acid stimulation and in the stress response and cell death (37).

OCA is a semisynthetic hydrophobic bile acid (BA) analogue that is a highly selective and potent FXR agonist. OCA is approved for the treatment of primary biliary cholangitis and is being evaluated for treatment of other liver disorders. In this study treatment of mice with OCA for 4 days after CBDL preserved *Abcb11* mRNA and protein levels and suppressed miR-199a-5a expression at levels found in sham-operated mice. Although OCA treatment led to attenuation of liver injury, FXR-mediated bile acid signaling will have ubiquitous effects on other pathways including those involving other transporters, inflammation, the microbiome, and fibrogenesis (38, 39).

There has been considerable effort devoted to elucidating the mechanism underlying miRNA biogenesis and target gene regulation. However, much less known is about the regulation of microRNA genes themselves at the levels of their promoters (40, 41). Transcriptional regulation of microRNAs by FXR has been demonstrated. For example, an FXR-responsive element has been identified in the miR-29a promoter, which inhibits the production of several extracellular matrix proteins in the liver (42). The mechanism for transcriptional repression of an miRNA by FXR usually occurs indirectly by inducing the expression of small heterodimer partner (SHP), an orphan nuclear receptor, and transcriptional repressor (43). In other cases, the mechanism for inhibition of microRNA expression by FXR has not been defined. For example, activation of FXR by its ligand reduced the level of miR-199a-3p in HepG2 cells, but the pathway for this effect was not examined (44). miR-199a-3p is generated from the same stem-loop RNA as miR-199a-5p and is involved in the regulation of similar biological processes. In the current study a novel regulatory mechanism demonstrates that FXR was a strong negative regulator of miR-199a-5p expression. In keeping with this notion, miR-199a-5p was increased sixfold in FXR^hep−/−^ mice compared with wild-type mice. Computational mapping of the promoter region of the gene encoding miR-199a-5p, *miR-199a-2*, identified a well-conserved FXR binding site (FXRE), which was validated by multiple *in vitro* studies. Enhanced specific binding of FXR/RXR to the FXRE in the *miR199a-2* promoter occurred in the presence of the FXR agonist GW4064. Moreover, in the livers of mice after CBDL, the FXR agonist obeticholic led to retention of the transcriptional repressor NcoR1 at the FXRE locus of *miR-199a-2*. Overall, it is clear that FXR agonists in cholestasis act by directly transactivating genes such as *ABCB11*, but our data adds to regulatory complexity of this process as FXR agonists also act by blocking an inhibitory pathway mediated by miR-199a-5p.

## Experimental procedures

### Animals

CBDL was performed on male C57BL6/J mice after 10 weeks of age, as previously described, using a protocol approved by the Institutional Animal Care and Use Committee of University of Colorado, Denver, CO (21). All animals received humane care according to the criteria outlined in the *Guide for the Care and Use of Laboratory Animals* prepared by the National Academy of Sciences and published by the National Institutes of Health. Mice were housed in a temperature, humidity, and ventilation-controlled vivarium and kept on a 12-h light/dark cycle. In brief, laparotomy was performed on the mice, and the common bile duct ligated proximally and distally and severed in the middle. Serum bile acids were estimated by a kit from Trinity Biotech to document that successful cholestasis was achieved (data not shown). Sham surgery was done in which laparotomy and manipulation of the liver wereperformed, but the bile duct was not ligated. Mice were randomly divided into four groups: sham surgery control group, sham surgery group with cellulose treatment (vehicle group), CBDL group, and CBDL group treated with OCA (INT-747). The vehicle group was given methylcellulose by gavage once daily for 4 days, while the OCA group was treated at a dose of 30 mg/kg in methylcellulose by gavage once daily for 4 days. CBDL was done in the morning and gavage treatment in the evening of the same day. All mice were sacrificed on day 4, and samples collected at the end of study. OCA (INT-747) was a kind gift of Intercept Pharmaceuticals. For gavage, OCA was dissolved in 0.75 to 1.0 ml of freshly prepared methylcellulose (1%), with equal volumes of vehicle administered to controls.

FXR hepatocyte specific knockout (KO) mouse livers were a kind gift of Bo Kong, PhD and Grace L. Guo, PhD, Department of Pharmacology and Toxicology, School of Pharmacy, Rutgers University, Piscataway, New Jersey. FXR hepatocyte-specific knockout mice (FXR^hep−/−^) have been described previously (45). In brief, mice with “floxed” FXR (FXR^flox\flox^) were crossed with FXR^flox\flox^ mice carrying albumin promoter-driven Cre (Cre^+/−^) to generate FXR^flox\flox^\Cre^−/−^ (wild-type mice, herein referred to as WT) and FXR^flox\flox^\Cre^+/−^ (hepatocyte-specific FXR KO mice, herein referred to as FXR^hep−/−)^ mice. Both WT and FXR^hep−/−^ were on a C57BL/6J genetic background and were maintained in pathogen-free animal facilities in the Laboratory of Animal Research under a standard 12-h light–dark cycle with food and water provided ad libitum. All the protocols and procedures were approved by the Institutional Animal Care and Use Committee.

### Blood and tissue sampling

Heparinized blood samples were collected after puncture of the aorta, spun at 3500 rpm for 10 min, frozen in liquid nitrogen, and stored at −80 °C. Liver tissue was frozen in liquid nitrogen and stored at −80 °C for Western blot (WB) analysis and quantitative reverse transcription–polymerase chain reaction (RT-qPCR) analysis.

### Human liver samples

A pediatric liver biobank at the University of Colorado provided liver biopsy samples from three infants with biliary atresia at the time of Kasai portoenterostomy and from three normal livers. Our institutional review board approved use of residual tissue from clinically indicated liver biopsies and from unused pediatric donor tissue from reduced-size liver transplants.

### Cells and cell culture

The human hepatoma cell line Huh-7 was cultured in Roswell Park Memorial Institute (RPMI)1640 medium with fetal bovine serum (FBS) and antibiotics, as previously described (11, 21). The cell lines were obtained from the American Tissue Culture Collection and were grown in 5% CO2 in a humidified incubator maintained at 37°.

### Serum liver enzyme quantification

Biochemical assessment of liver injury in CBDL and CBDL + OCA-treated mice. On the day of sacrifice, mice were anesthetized with i.p. ketamine/xylazine and blood collected from the retro-orbital plexus and liver removed, placed in formalin or snap frozen in liquid nitrogen, and subsequently stored at −80 °C until analyzed. Serum aspartate aminotransferase (AST), alanine aminotransferase (ALT), alkaline phosphatase(ALP), and total Bilirubin were analyzed at the University of Colorado Hospital Clinical Chemistry Laboratory from coded serum samples.

### Histological analysis

Liver tissues were removed at sacrifice, formalin-fixed, paraffin-embedded, and sectioned at 5 microns, then stained with Hematoxylin and Eosin, using standard histologic techniques. Histologic images were captured on an Olympus BX51 microscope equipped with a four-megapixel Macro fire digital camera (Optronics) using the Picture Frame Application 2.3 (Optronics). All images were cropped and assembled using Photoshop CS2 (Adobe Systems, Inc). Images were then imported into Slidebook, five images per animal, three to seven animals per group. To ensure rigor and reproducibility, coded slides were examined and scored in a blinded fashion by a trained experimental pathologist (DJO).

### Materials

The anti-BSEP/ABCB11 (PAB4697) antibody was purchased from Abnova. Antibodies to MRP2/ABCC2 (ab3373) were obtained from Abcam. Anti-FXR/NRIH4 (252165) antibodies were purchased from Abbiotec. The anti-β-actin (A5316) antibody was purchased from Sigma/Aldrich. Cell culture media, FBS, and Lipofectamine 2000 were obtained from Invitrogen. The anti-β-actin (A5316) antibody was purchased from Sigma/Aldrich. Cell culture media, FBS, and Lipofectamine 2000 were obtained from Invitrogen. miR mimics, inhibitors, and negative controls were obtained from Exiqon/Qiagen. All other chemicals were from Sigma or Fisher Scientific unless otherwise stated.

### Plasmid constructs

The 3′UTR luciferase plasmid for the human and mouse *pMIR/ABCB11/Abcb11* was a generous gift from Dr M. Ananthanarayanan (Scientific Review Officer at The National Institutes of Health). All of the positive clones containing 3′UTR inserts were verified by restriction enzyme mapping and sequenced using the ABI automated DNA sequencer model 377.

### Bioinformatics

An *in silico* search for possible miRNA-binding sites in the 3′ UTR of the *ABCB11/Abcb11* (BSEP) gene was done by using miRANDA (Memorial Sloan-Kettering Cancer Center), DIANA-microT-CDS (Biomedical Science Research Center Alexander Fleming), and miRBase (Faculty of Life Science, University of Manchester, Manchester, UK) (46, 47).

*miR-199a-2* is the gene encoding miR-1999a-5p (48). The putative promoter region of mouse and human *miR-199a-2* (miRPR-199a-2) was previously predicted using a genome-wide algorithm for miRNA gene promoters. Promoter sequence of the miR-199a-2 gene (miRPR-199a-2) and the putative binding sites of several transcriptional factors were detected by TRANSFAC Alibaba 2.1 and Genomatrix software. There is a putative FXR-binding site in both human and mouse miRPR-199a-2.

### Transient transfection with miR-199a-5p and let-7a-5p

To investigate the effect of miR-199a-5p on ABCB11 expression, Huh-7 was seeded in 12-well plates (8 × 10^4^ cells/ml). After 24 h cells were transfected with 100 nM has-miR VANA miRNA mimics or anti-miR miRNA inhibitors and corresponding negative controls (Exiqon/Qiagen) by using Lipofectamine RNAiMax diluted in Opti-MEM I (both purchased from Invitrogen) at a final concentration of 3 mM. After 8 h of incubation, the transfection medium was replaced with fresh complete growth medium. At 48 and 72 h after transfection, total mRNA and protein were isolated by use of RNAeasy Mini Kit (Qiagen) (Life Technologies) and M-PER (Mammalian Protein Extraction Reagent, Pierce Scientific) containing proteolytic and phosphatase inhibitor mixture (Sigma-Aldrich). mRNA and protein were quantified in three independently performed experiments.

To examine miRNA-dependent expression of FXR target genes, Huh-7 cells were treated with 1 μM of the FXR agonist GW4064 (Tocris Bioscience) for 24 h before transfection of miRNA mimics and inhibitors. Data were merged from four experiments. Corresponding negative controls (Exiqon/Qiagen) were transfected simultaneously with the miRNA mimics for 48 h.

### Western blotting after mimic, inhibitor, or negative control transfection

To examine the effect of miR-199a-5p and let-7a-5p overexpression on proteins levels, Huh-7 cells were plated in 6-well plates at a density of 1 × 10^5^ cells/well 24 h prior to transfection. The following day, the cells were transfected with mimics, inhibitors, or negative controls at a concentration of 50 and 100 nm after complexing with Lipofectamine RNAiMax at a ratio of 1:3 (μl/μl) in Opti-MEM medium. The cells were fed with RPIM 1640 medium after 24 h. Forty-eight and seventy-two hours after transfection, the cells were lysed with 150 μl of M-PER (Mammalian Protein Extraction Reagent, Pierce Scientific) containing proteolytic and phosphatase inhibitor mixture (Sigma). Western blotting was carried out after protein quantitation.

### Western blotting analyses

Protein extracts of Huh-7 cells transiently transfected with control, mimics, and inhibitors were lysed for 15 min on ice in 150 μl of M-PER (Pierce Scientific) containing protease and phosphatase inhibitor mixtures. Cell debris was removed by centrifugation at 16,000 rpm (21,130*g*) for 15 min at 4 °C in an Eppendorf microcentrifuge. Protein concentration in the supernatant was estimated using BSA as standard (Bio-Rad). In total, 50 to 75 μg of protein in 1 × Laemmli buffer was loaded per lane of a 4 to 20% Mini-Protean TGX precast gels (Bio-Rad) and run at 200 V for 40 min. Prestained Precision Plus Dual Color Protein standards (Bio-Rad) were also run on the same gels to estimate protein molecular size. The fractionated proteins were blotted using a Bio-Rad Semidry blotter (Trans-Blot SD semidry blotter cell) to precut PVDF membranes (Immunoblot PVDF membrane, 7.0 × 8.5 cm) in Tris glycine/methanol (20%) buffer at 20 V for 90 min. Following protein transfer, the blots were blocked with 5% Nonfat Dry Blot Omniblok (American Bioanalytical) in 1 × blocking buffer (diluted from 10 × Tris-buffered saline with 0.5% Tween 20, KPL). Following blocking, incubation with primary antibodies (to BSEP, MRP2, NCOR1, SMRT, and β-actin) with secondary peroxidase-conjugated antibodies and the washing steps were performed as previously described. Signals developed with clarity western ECL substrate (Bio-Rad) were quantitated using Bio-Rad ChemiDoc System.

### Total RNA isolation and real-time PCR assays

Total RNA was isolated from cells as denoted in the figure legends using the RNAeasy Mini Kit (Qiagen) according to the manufacturer's instructions. Quantitation of RNA was done using Nanodrop 2000. cDNA synthesis was carried out using the Affinity Script Multi Temperature cDNA Synthesis Kit (Agilent Biotechnologies) on 2 μg of total RNA as per the manufacturer's directions. The cDNA was diluted tenfold and 5 μl of the diluted cDNA (100 ng) per well was utilized for qPCR using SYBR Green as the detection method employing Power SYBR Green Gene expression master mixtures. Oligonucleotide primers used in the SYBR Green qPCR assays will be provided on request. In SYBR Green assays, normalization was achieved using 36B4 as a housekeeping control and β-Actin levels assays. All qPCRs were done using an ABI Quant Studio 7 Flex Real-Time PCR System machine located in our lab. Relative expression was calculated using the comparative *C*_*T*_ method (ΔΔ*C*_*T*_ method) as per the manufacturer's instructions (Applied Biosystems). Primer sequences not included here are available on request.

### siRNA-mediated knockdown of FXR (NR1H4)

ON-TARGET plus siRNA human NRIH4 used in this study was purchased from Dharmacon, Horizon discovery. Huh-7 cells were plated in six-well plates (1 × 10^6^ cells/well) and incubated 2 days later with 50 nM siRNA using TransIT-TKO (Mirus Bio LLC) at a ratio of 1:1 according to the manufacturer's instructions. Six hours later, medium was added to the wells, and 24 h later, spent medium was replaced with fresh RPMI 1640. Forty-eight hours later, total RNA and miRNA were prepared using the RNAeasy Mini Kit and miRNeasy mini kit (Qiagen). Real-time PCR analysis was done after conversion of mRNA and miRNA into cDNA.

### Luciferase reporter gene assays—human ABCB11 3′UTR

A 1440 bp DNA amplicon of the 3′ UTR region of the human *ABCB11* mRNA with 199a-5p target site was obtained by RT-PCR using specific oligonucleotides forward (CCAATGCAAGAATCTCAGACACA) and reverse (GACCTCTTTTCTAAGATTGGCCAAT). This fragment was subcloned into the pMIR- REPORT luciferase vector (Applied Biosystems) resulting in a CMV-driven expression construct Luc-*ABCB11*-3′ UTR. Huh-7 cells were plated in 24-well plates at a density of 1 × 10^5^ cells/well 24 h before transfection. miRNA mimics and inhibitors and negative controls 1 and 2 (Exiqon) were transfected at a final concentration of 50 nm in combination with Luc-*ABCB11*-3′ UTR (0.25 μg/well) in Opti-MEM complexed to Lipofectamine 2000 at a ratio of 1:3 (μg:μl) as per the manufacturer's instructions. We used LNA-modified mimics and inhibitors (Exiqon) because the unmodified reagents showed nonspecific effects. Twenty-four hours later, the medium was changed to RPMI 1640 medium. Forty-eight hours after transfection cell lysates were prepared in 250 μl of 1 × Passive Lysis Buffer (Promega). Firefly and *Renilla* luciferase activities were assayed in 50 μl of the lysate using the Dual Luciferase Kit (Promega) as per the manufacturer's instructions using a luminometer (Promega). Relative luciferase activities were reported after normalization of the individual values to *Renilla* luciferase (49).

Point mutations were introduced into the human *ABCB11* 3′UTR using a Quick-Change II XL Site-Directed Mutagenesis Kit (Agilent) and appropriate mutant oligos. Mutants were confirmed by nucleic acid sequencing and those plasmids were used for the transfection (WT hABCB11 5′aagatgtaaaaACACTGGatccttctg3′ and hABCB11 MU-5′- aagatgtaaaaGACCGAAatccttctg-3′).

### Luciferase reporter gene assays *miR199a-2* promoter

A 1420 bp DNA amplicon of the promoter region of the human miR-199a-2, which included the FXR (NR1H4) target site, was obtained by Gene Art Invitrogen by Thermo Fisher Scientific (Life Technologies). This fragment was subcloned into the PGL3 basic luciferase vector (Promega) resulting in a CMV-driven expression construct Luc-199a-2 promoter. Point mutations were introduced to remove the FXRE from the human *miR199a-2* promoter using a Quick-Change II XL Site-Directed Mutagenesis Kit (Agilent) and appropriate mutant oligos. Mutants were confirmed by nucleic acid sequencing.

Huh-7 cells were plated at a concentration of 1 × 10^5^ cells/well in 24-well plates for 48 h. Cells were transfected at day 0 with the human 199a-2 promoter at 0.5 μg/well (in triplicate/per group) and also cotransfected with 50 ng of *FXR/RXRα* expression plasmids in OPTI-MEM (Invitrogen). Transfections were carried out using TransIT-LT (Mirus Bio) at a DNA:TransIT ratio of 1:3. FXR ligand GW4064 (1 μm) was added after 24 h transfection and luciferase activities were measured 24 h later using the Promega kit (Promega). Normalization of transfection efficiencies in the different wells was achieved by cotransfection with pCMV-β galactosidase and the assay of galactosidase activity.

### Electrophoretic mobility shift assays (EMSAs)

EMSA was performed as reported previously by us (50). In brief, FXR and RXR cDNAs (5 μg/dish) was transfected into Huh-7 cells (5 × 10^6^ cells/100-mm dish) in three dishes using TransIT-LT1 at a DNA/TransIT-LT1 ratio of 1:3. Control untransfected cells were left in RPMI 1640 medium. TransIT-LT1 was replaced with RPMI 1640 medium 1 day later, and 3 days later nuclear extracts were prepared using NE-PER from Thermo Fisher Scientific according to the manufacturer's directions. Nuclear extracts were stored in aliquots at −80 °C until used. The oligonucleotide probe (human *miR199a-2* FXRE) for the EMSAs was end-labeled with [γ-^32^P]ATP (3000 or 6000 mCi/mmol) by T4 polynucleotide kinase. EMSA assays with 10 μg of Huh-7 nuclear extracts were added to 20 μl of binding reaction containing 12 mM HEPES (pH 7.9), 60 mM KCl, 4 mM Tris-HCl, 5% glycerol, 1 mM EDTA,1 mM dithiothreitol, 1 μg of polydeoxyinosinic-deoxycytidylic acid, 1 μg of salmon sperm DNA, and 5 × 10^4^ cpm of probe on ice for 45 min. As a control, the probe was also incubated with the same amount of untransfected Huh-7 nuclear extract. In competition assays, unlabeled wild-type or mutant oligonucleotides were added to the reaction 15 min before the addition of the probe. DNA–protein complexes were resolved on 4% native polyacrylamide gel electrophoresis containing 0.5 × TBE (0.89 m Tris, 0.89 m boric acid, 0.02 m disodium EDTA for 10 × TBE). The gel was dried and exposed to X-ray film for varying lengths of time until a suitable image was obtained.

For *in vitro* experiments, a cDNA encoding human FXR and RXR was transcribed and translated using the TNT-coupled reticulocyte lysate system (Promega) according to the manufacturer’s instructions. Five microliters of RXRα and FXR cDNA-programmed lysates alone or together were added to 20 μl of binding reaction mention above. As a control, the probe was also incubated with the same amount of unprogrammed TNT lysate. In competition assays, unlabeled wild-type or mutant oligonucleotides were added to the reaction 15 min before the addition of the probe. A super-shifted band was demonstrated with the addition of an FXR antibody.

### Chromatin immunoprecipitation analysis of mouse liver

ChIP assays from the mouse liver were done by a combination of protocols previously used by us and manufacturer’s instructions using Magna ChIP G Tissue kit from Millipore Sigma (23, 50). In brief, mouse livers were sliced to small pieces and then incubated with 1% formaldehyde to cross-link proteins to genomic DNA in cells. Following this incubation, excess formaldehyde was quenched by incubation with glycine. Chromatin was prepared from cross-linked livers after isolation of nuclei. Chromatin was sonicated with the appropriate power setting to shear DNA to approximately ∼500 bp fragments for use in ChIP. After sonication, the fragmented DNA was diluted in ChIP dilution buffer and preabsorbed with Protein G Sepharose/salmon sperm DNA (Millipore Sigma) for 1 h at 4 °C. Then 5% of the chromatin was removed and saved as input. It was then incubated overnight at 4 °C with 3 to 5 μg of the appropriate (Fxr, Ncor1 and Smrt) antibodies or normal mouse IgG (control). Antibody–chromatin complexes were captured by incubation with Protein G Sepharose and centrifuged. Reversal of protein cross-linking and proteinase K digestion, followed by purification of the DNA, was then achieved. An aliquot of the DNA (2 μl) was used in a PCR (standard and quantitative) reaction using specific primers flanking the FXRE of mouse *miR199a-2* promoter. Primers flanking a site distant from the FXRE sites were used as negative controls. PCR products were run in a 2% agarose gel and stained with ethidium bromide to confirm the amplicon size.

### Statistical analysis

Data are expressed as mean ± SE. A two-tailed paired Student *t* test was used in comparing two groups. For multiple comparisons, an ordinary one-way ANOVA analysis followed by post hoc Tukey’s multiple comparison tests was done by using Prism 8 software. *p* < 0.05 was considered statistically significant. All experiments using cultured cells or mouse livers.

## Data availability

All data are in the article and Supporting information are available upon request from the authors: Natarajan Balasubramaniyan (nata.bala@cuanschutz.edu) and Frederick J. Suchy (frederick.suchy@childrenscolorado.org) at Department of Pediatrics, Digestive Health Institute, Children’s Hospital Colorado, University of Colorado School of Medicine.

## Supporting information

This article contains supporting information.

## Conflict of interest

F. J. S., N. B., D. J. O., and M. W. D. have declared no conflicts of interest. R. J. S has consulted with Mirum, Albireo, and Alexion during the past 12 months.

## Supporting information

Table S1 FXR agonist OCA treatment ameliorates hepatic injury and reduces cholestasis during murine CBDL. CBDL mice were treated with OCA by gavage on days 1 through 4 and serum measures of hepatic injury and cholestasis were obtained at 4 days and compared among several experimental groups: Sham, Sham-Cellulose, CBDL, and CBDL-OCA. OCA treatment reduced all elevated markers levels. (Row 2) Serum alanine aminotransferase (ALT), (Row 3) Serum aspartate aminotransferase (AST), (Row 4) Serum alkaline Phosphatase (ALP), and (Row 5) Total Bilirubin. Data are means ± SEM, n = 3 to 4 animals per group. Statistical analysis was performed using one-way analysis of variance and Tukey’s correction for multiple comparisons. A two-tailed paired Student *t* test was used in comparing two groups. ∗ *p* ≤ 0.05.

Figure S1 *Abcb11* and miR199a-5p expression in mice 4, 7, and 14 days after CBDL. Statistical analysis was performed using one-way analysis of variance and Tukey’s correction for multiple comparisons. A two-tailed paired Student *t* test was used in comparing two groups. ∗ *p* ≤ 0.05.

Figure S2 Liver sections from Sham, Sham+Cellulose, CBDL, and CBDL+OCA treated mice were stained with hematoxylin and eosin, as described in Experimental procedures. There is marked reduction in areas of necrosis in mice treated with OCA. Representative images are shown of at least three mice per treatment. Representative images are shown of at least three mice per treatment. The size bar is 500 microns.
