# Supplementary material for: miR-199a-5p inhibits the expression of ABCB11 in obstructive cholestasis
Source: J Biol Chem. 2021 Nov 12;297(6):101400. doi: 10.1016/j.jbc.2021.101400 (PMC8665360; doi:10.1016/j.jbc.2021.101400)
Supplement: Table S1 — FXR agonist OCA treatment ameliorates hepatic injury and reduces cholestasis during murine CBDL. CBDL mice were treated with OCA by gavage on days 1 through 4 and serum measures of hepatic injury and cholestasis were obtained at 4 days and compared among several experimental groups: Sham, Sham-Cellulose, CBDL, and CBDL-OCA. OCA treatment reduced all elevated markers levels. (Row 2) Serum alanine aminotransferase (ALT), (Row 3) Serum aspartate aminotransferase (AST), (Row 4) Serum alkaline Phosphatase (ALP), and (Row 5) Total Bilirubin. Data are means ± SEM, n = 3 to 4 animals per group. Statistical analysis was performed using one-way analysis of variance and Tukey’s correction for multiple comparisons. A two-tailed paired Student t test was used in comparing two groups. ∗ p ≤ 0.05. [file mmc1.pdf]

Serum ALT, AST, ALP and Total Bilirubin concentrations After CBDL+OCA treatment

Groups	ALT(U/L)	AST(U/L)	ALP(U/L)	T BILIRUBIN (mg/dl)
Sham-4d	30.4±3.9	75.2±7.5	48.7±4.4	0.5±0.20
Sham+Cellulose-4d	31.0±5.2	70.4±9.2	47.3±6.2	0.4±0.18
CBDL-4d	305.0±56.2*	526.4±53.0*	415.3±30.2*	17.2±6.4*
CBDL+OCA-4d	148.8±11.0*	242.2±25.4*	201±12.1*	4.8±1.5*
