# Supplementary figures and images for: miR-199a-5p inhibits the expression of ABCB11 in obstructive cholestasis
Source: J Biol Chem. 2021 Nov 12;297(6):101400. doi: 10.1016/j.jbc.2021.101400 (PMC8665360; doi:10.1016/j.jbc.2021.101400)

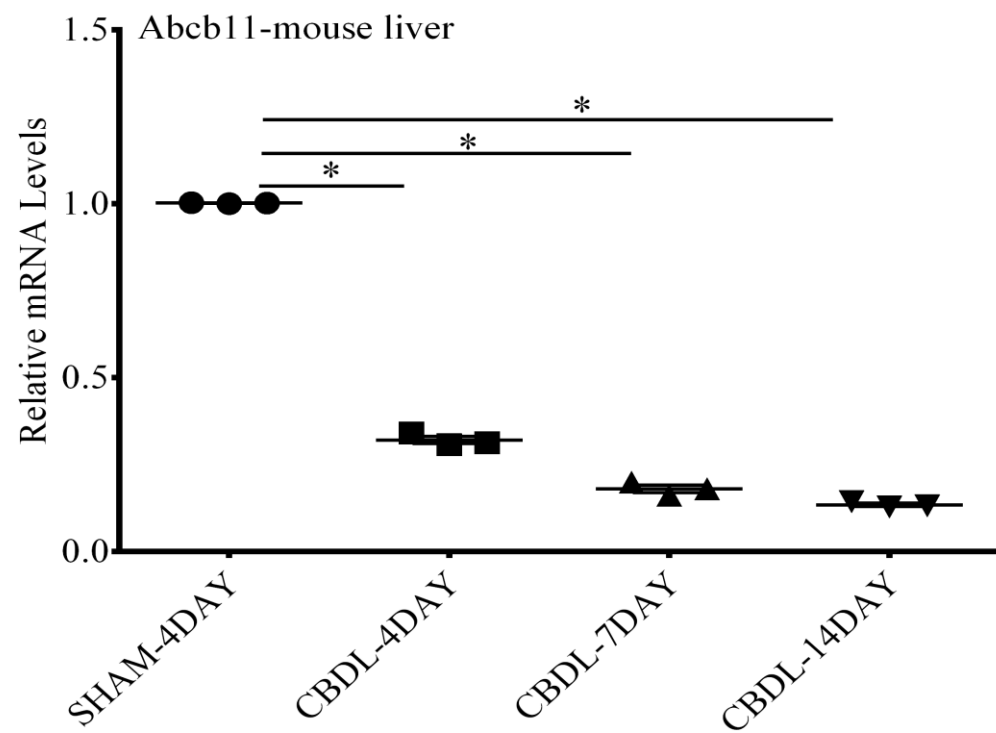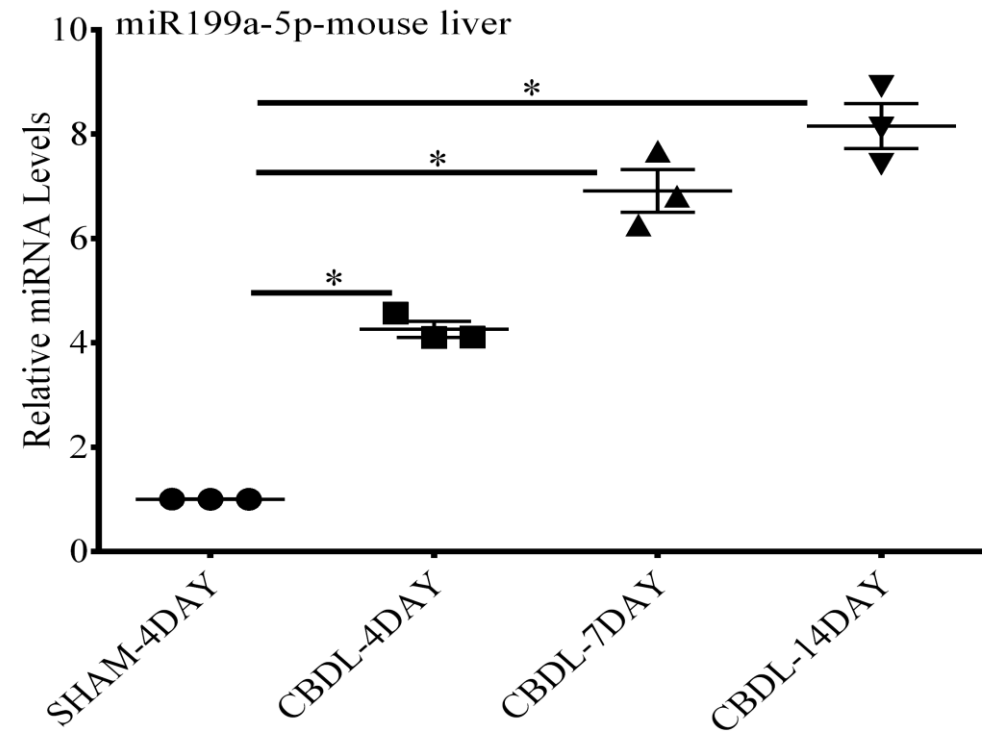

Supplement: Figure S1 — Abcb11 and miR199a-5p expression in mice 4, 7, and 14 days after CBDL. Statistical analysis was performed using one-way analysis of variance and Tukey’s correction for multiple comparisons. A two-tailed paired Student t test was used in comparing two groups. ∗ p ≤ 0.05. [file mmc2.pdf]

**Sham**

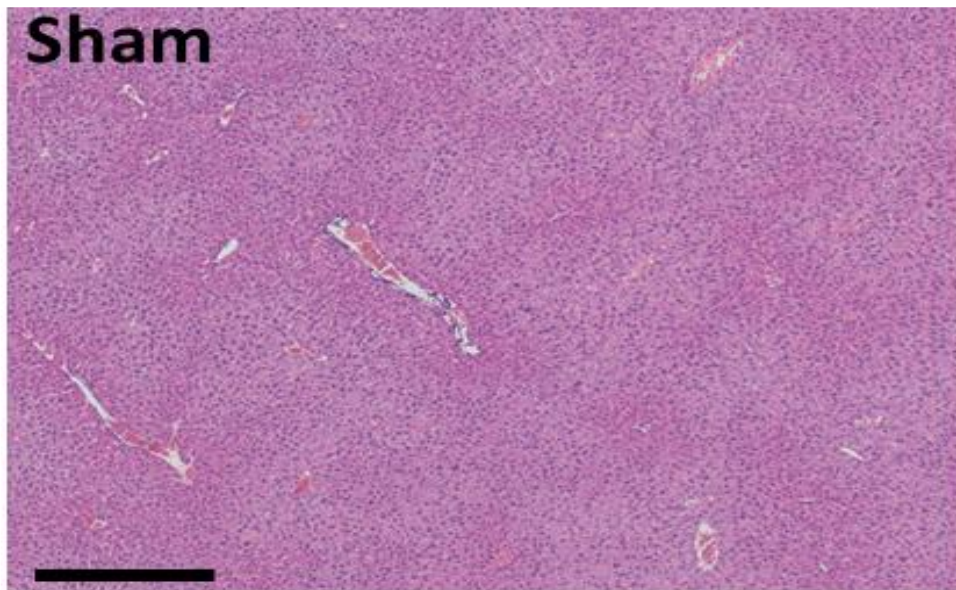

**Sham + Cellulose**

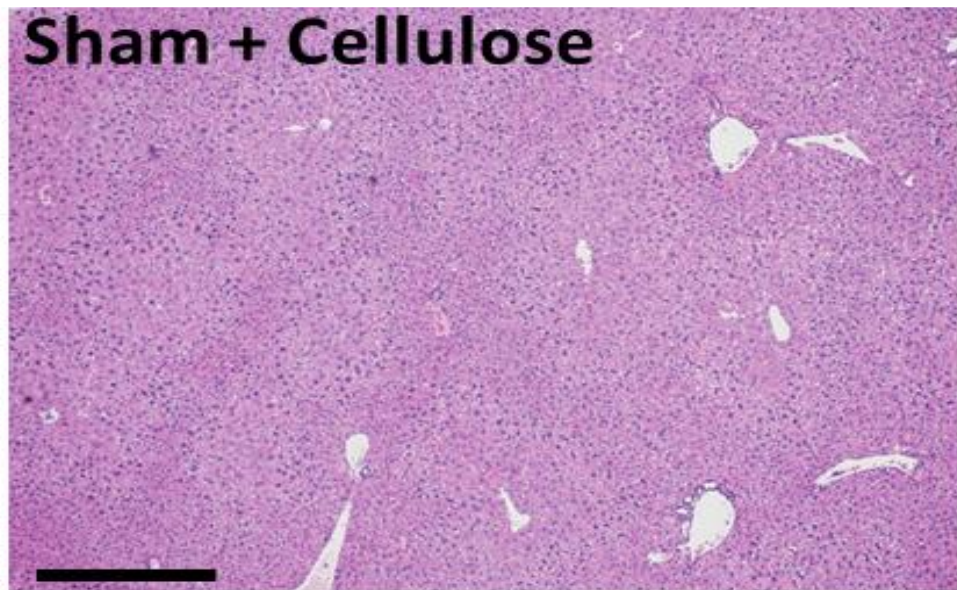

**CBDL**

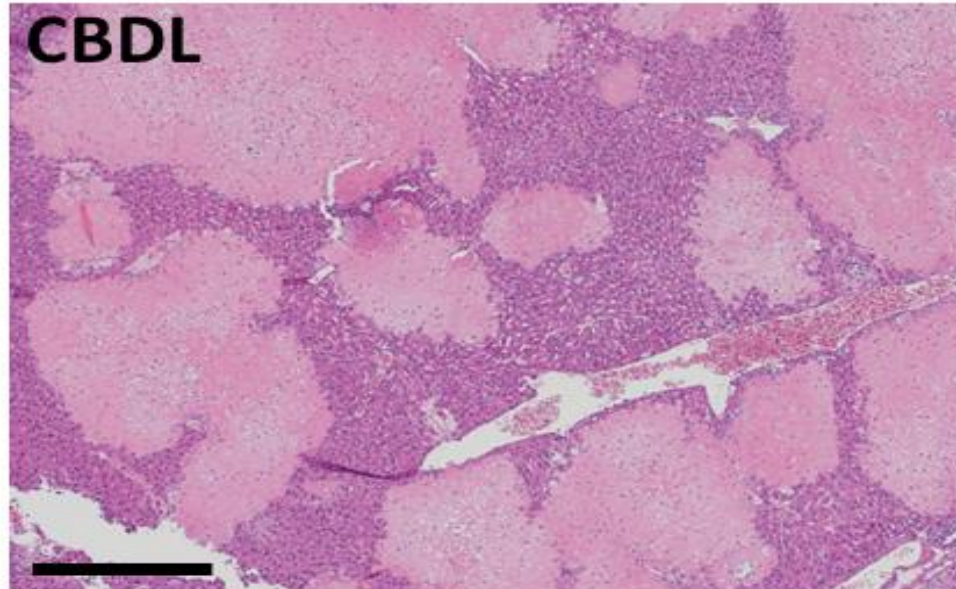

**CBDL + OCA**

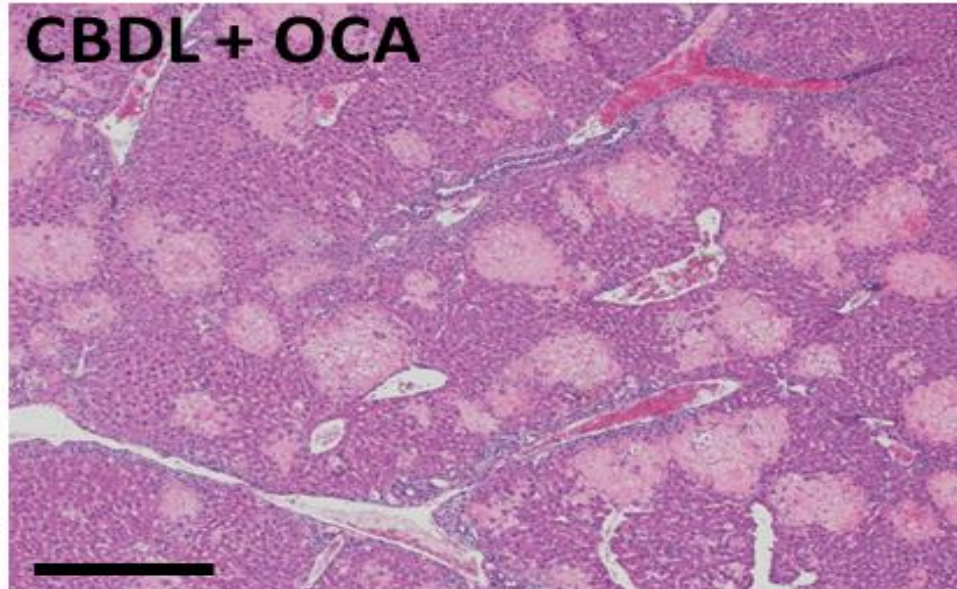

Supplement: Figure S2 — Liver sections from Sham, Sham+Cellulose, CBDL, and CBDL+OCA treated mice were stained with hematoxylin and eosin, as described in Experimental procedures. There is marked reduction in areas of necrosis in mice treated with OCA. Representative images are shown of at least three mice per treatment. Representative images are shown of at least three mice per treatment. The size bar is 500 microns. [file mmc3.pdf]
